# Surveillance of Extended-Spectrum Beta-Lactamase-Producing *Escherichia coli* in Dairy Cattle Farms in the Nile Delta, Egypt

**DOI:** 10.3389/fmicb.2016.01020

**Published:** 2016-07-04

**Authors:** Sascha D. Braun, Marwa F. E. Ahmed, Hosny El-Adawy, Helmut Hotzel, Ines Engelmann, Daniel Weiß, Stefan Monecke, Ralf Ehricht

**Affiliations:** ^1^Alere Technologies GmbHJena, Germany; ^2^InfectoGnostics Research CampusJena, Germany; ^3^Department of Animal Hygiene and Zoonoses, Faculty of Veterinary Medicine, Mansoura UniversityMansoura, Egypt; ^4^Institute of Bacterial Infections and Zoonoses, Friedrich-Loeffler-InstitutJena, Germany; ^5^Department of Poultry Disease, Faculty of Veterinary Medicine, Kafrelsheikh UniversityKafr El-Sheikh, Egypt; ^6^Institute for Medical Microbiology and Hygiene, Technical University of DresdenDresden, Germany

**Keywords:** ESBL, carbapenemases, *Escherichia coli*, Egypt, dairy cattle, microarray, genotype, CRE

## Abstract

**Introduction:** Industrial livestock farming is a possible source of multi-resistant Gram-negative bacteria, including producers of extended spectrum beta-lactamases (ESBLs) conferring resistance to 3rd generation cephalosporins. Limited information is currently available on the situation of ESBL producers in livestock farming outside of Western Europe. A surveillance study was conducted from January to May in 2014 in four dairy cattle farms in different areas of the Nile delta, Egypt.

**Materials and Methods:** In total, 266 samples were collected from 4 dairy farms including rectal swabs from clinically healthy cattle (*n* = 210), and environmental samples from the stalls (*n* = 56). After 24 h pre-enrichment in buffered peptone water, all samples were screened for 3rd generation cephalosporin-resistant *Escherichia coli* using Brilliance™ ESBL agar. Suspected colonies of putatively ESBL-producing *E. coli* were sub-cultured and subsequently genotypically and phenotypically characterized. Susceptibility testing using the VITEK-2 system was performed. All suspect isolates were genotypically analyzed using two DNA-microarray based assays: CarbDetect AS-1 and *E. coli* PanType AS-2 kit (ALERE). These tests allow detection of a multitude of genes and their alleles associated with resistance toward carbapenems, cephalosporins, and other frequently used antibiotics. Serotypes were determined using the *E. coli* SeroGenotyping AS-1 kit (ALERE).

**Results:** Out of 266 samples tested, 114 (42.8%) ESBL-producing *E. coli* were geno- and phenotypically identified. 113 of 114 phenotypically 3rd generation cephalosporin-resistant isolates harbored at least one of the ESBL resistance genes covered by the applied assays [*bla*CTX-M15 (*n* = 105), *bla*CTX-M9 (*n* = 1), *bla*TEM (*n* = 90), *bla*SHV (*n* = 1)]. Alarmingly, the carbapenemase genes *bla*OXA-48 (*n* = 5) and blaOXA-181 (*n* = 1) were found in isolates that also were phenotypically resistant to imipenem and meropenem. Using the array-based serogenotyping method, 66 of the 118 isolates (55%) could be genotypically assigned to O-types.

**Conclusion:** This study is considered to be a first report of the high prevalence of ESBL-producing *E. coli* in dairy farms in Egypt. ESBL-producing *E. coli* isolates with different underlying resistance mechanisms are common in investigated dairy cattle farms in Egypt. The global rise of ESBL- and carbapenemase-producing Gram-negative bacteria is a big concern, and demands intensified surveillance.

## Introduction

Extended-spectrum beta-lactamases (ESBLs) are mainly plasmid-encoded enzymes providing resistance to 3rd generation (3G) cephalosporins. These enzymes can be produced by a variety of different bacteria, such as *Enterobacteriaceae* or non-fermenting bacteria (Bradford, 2001; Giamarellou, 2005; Rawat and Nair, 2010; Shaikh et al., 2015). The most frequently found ESBL-producing species is *Escherichia coli* which often causes urinary tract infections, pneumonia or even sepsis in humans (Abraham et al., 2012). ESBL-producing *E. coli* has been broadly recognized in veterinary medicine as causative agents of mastitis in dairy cattle since the 2000s (Brinas et al., 2003; Haftu et al., 2012), but only a few studies exist that investigated the prevalence of ESBL-producing bacteria in livestock, showing their existence in sick and/or healthy cattle (Valentin et al., 2014; Dahms et al., 2015).

Unfortunately, there is no legislation in Egypt regulating the use of antibiotics (Dahshan et al., 2015). Antimicrobials such as tetracycline, quinolones, and beta lactams are still used in Egypt for growth promotion in animal feed and by veterinarians for the treatment and prevention of zoonotic diseases (WHO, 2013).

The CTX-M beta-lactamases, named for their greater activity against cefotaxime, are the most frequently detected ESBLs in livestock, and have been reported from different food-producing animals (Schmid et al., 2013; Brolund, 2014; Hansen et al., 2014). These animals also represent a source and/or a reservoir for ESBL-producing *E. coli* (Carattoli, 2008). Several studies indicate that these resistance genes are disseminated through the food chain or via direct contact between humans and animals (Schmid et al., 2013; Dahms et al., 2015). Data on ESBL-producing bacteria in food animals from Egypt are very limited. Therefore, the current study was conducted on four dairy cattle farms in different districts of Northern Egypt to assess the prevalence of ESBL-producing *E. coli* in dairy cattle and their environment.

## Materials and methods

### Farm description and sampling

**Figure 1:**
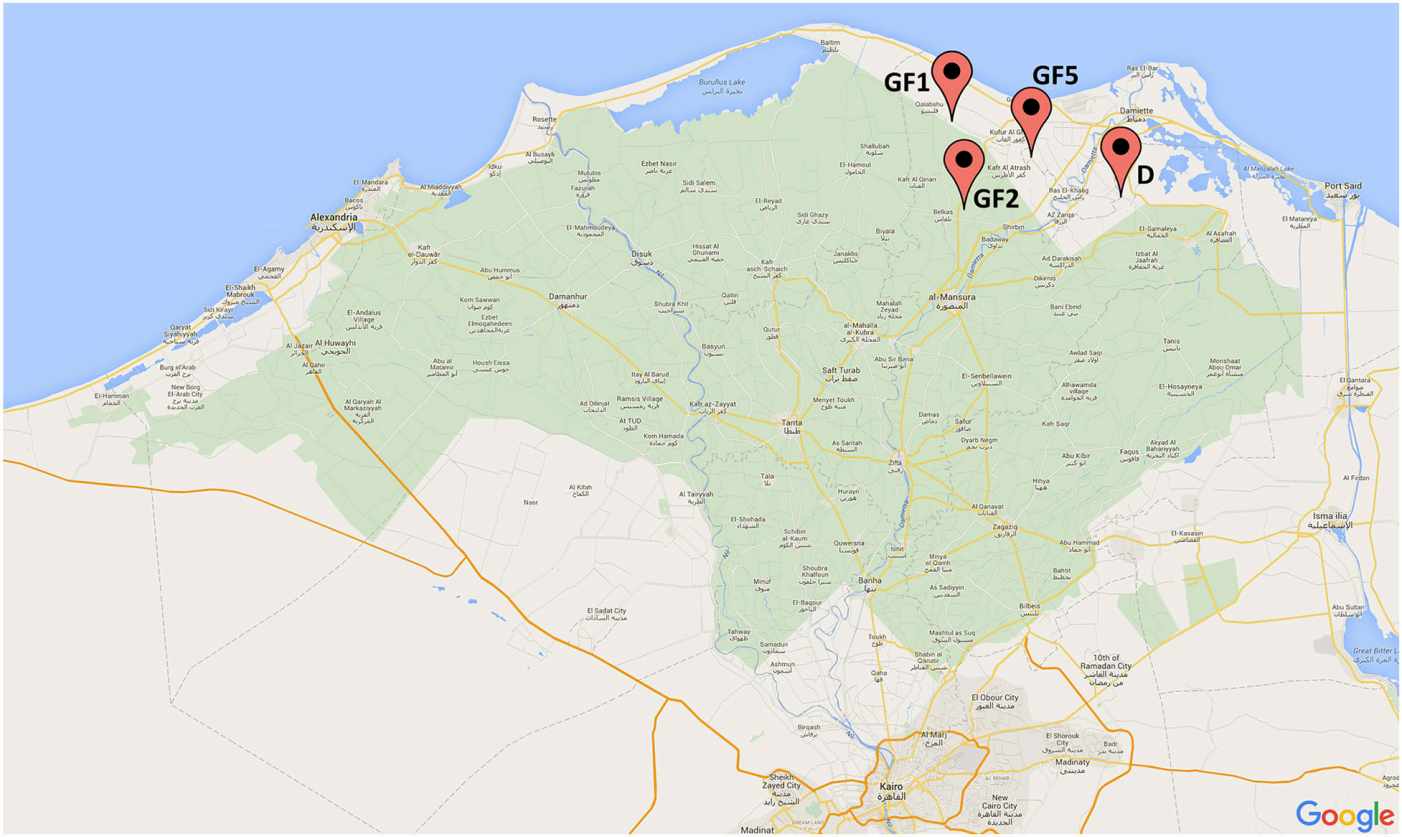
**Map of Nile Delta, Egypt with all locations of the dairy cattle farms from which the samples were collected (GF-Gamasa, D-Damietta)**.

In 2014, four dairy farms, three in Gamasa (GF1, GF2, GF5), and one in Damietta (D), were investigated (Figure 1). These farms were located in Nile Delta, Egypt in two different governorates (Damietta; Latitude N. 31⋅19′, Longitude E. 31⋅81′ and Dakahlia Latitude N. 31⋅25′, Longitude E. 31⋅32′). The herd size ranged from 400, 600, 650, and 800 in GF1, GF2, GF5, and D, respectively. The cattle enrolled in this study were between 2 and 10 years old. Half of the farms housed dairy cattle in free-stall barns and half of them in tie-stall barns. In total, 266 samples were collected from these farms. This included rectal swabs and milk samples from apparently healthy dairy cattle (*n* = 210), and environmental swab samples from water trough, feed and bedding (*n* = 56).

### Bacterial strains, isolation, identification, and genomic DNA extraction

All collected samples were enriched in buffered peptone water and cultivated on Brilliance™ ESBL agar (Oxoid, Wesel, Germany) for preliminary analysis for ESBL-producing *E. coli*. For further investigations, all suspected *E. coli* that grew on the selective medium were cultivated on tryptone yeast agar (Oxoid, Wesel, Germany). Presumptive characteristic *E. coli* isolates were identified by Gram staining and motility, and confirmed using a panel of biochemical tests (Triple Sugar Iron (TSI) agar, catalase, oxidase, H_2_S production and sugar fermentation) and API 20 E systems (bioMérieux, France; ISO, 2001). All confirmed isolates were subsequently re-tested using an automated microdilution technique (VITEK-2, bioMérieux, Nürtingen, Germany) that covered the following antibiotics: imipenem, meropenem, cefotaxime, ceftazidime, cefuroxime-axetil, cefuroxime, piperacillin/tazobactam, ampicillin/sulbactam, ampicillin, gentamicin, tobramycin, ciprofloxacin, moxifloxacin, tetracycline, tigecycline, co-trimoxazol, and fosfomycin (VITEK-2 test card: AST-N289). Susceptibility tests for chloramphenicol, kanamycin, streptomycin, erythromycin and colistin were not performed in this study.

Genomic DNA from clonal isolates was extracted using the DNeasy Blood & Tissue kit (Qiagen GmbH, Hilden, Germany) according to manufacturer's instructions. When necessary, DNA was concentrated to at least 100 ng/μl using a SpeedVac centrifuge (Eppendorf, Hamburg, Germany) at room temperature with 1400 rpm and for 30 min. Five microliters of recovered genomic DNA were used directly for biotin-labeling and subsequent hybridization.

### GenoSeroTyping and antimicrobial resistance genotype

For all ESBL-producing *E. coli*, the serotype was determined using the *E. coli* SeroGenoTyping AS-1 kit. The antimicrobial resistance (AMR) genotype was detected by the CarbDetect AS-1 kit and all other resistance genes were detected by the *E. coli* PanType AS-2 kit (Alere Technologies GmbH, Jena, Germany). The data were automatically summarized by the “result collector,” a software tool provided by Alere Technologies. An antibiotic resistance genotype was defined as a group of genes which have been described to confer resistance to a family of antibiotics (e.g., the genotype “*bla*CTX-M1/15, *bla*TEM” confers resistance to 3G cephalosporins) (**Table 2**).

### Multiplex labeling, hybridization, and data analysis

Extracted DNA was labeled by primer extension amplification using *E. coli* SeroGenoTyping AS-1, CarbDetect AS-1 or *E. coli* PanType AS-2 kits according to manufacturer's instructions. The procedure for multiplex labeling, hybridization and data analysis was described in detail by Braun et al. (2014). Briefly, internal labeling of the synthesized single stranded DNA resulted from the primer elongation of previously hybridized primers to the target genomic DNA, by using dUTP linked biotin as dideoxynucleotide triphosphate to be incorporated during synthesis. This procedure allowed site-specific internal labeling of the corresponding target region. The PCR protocol included 5 min of initial denaturation at 96⋅C, followed by 50 cycles with 20 s of annealing at 50⋅C, 40 s of elongation at 72⋅C, and 60 s of denaturation at 96⋅C (used device: Eppendorf Mastercycler gradient, Eppendorf, Hamburg, Germany). This reaction resulted in a multitude of specific linearly amplified, single-stranded, biotin-labeled DNA molecules for subsequent hybridization and detection using the DNA microarrays.

For hybridization procedures, the CarbDetect AS-1 and the *E. coli* PanType AS-2 kit were used according to manufacturer's instructions. CarbDetect ArrayStrips were placed in a thermomixer with an Alere ArrayStrip adapter (Quantifoil Instruments, Jena, Germany) and subsequently washed with 200 μl of deionized water at 50⋅C with 550 rpm for 5 min and with 100 μl hybridization buffer C1 at 50⋅C with 550 rpm for 5 min. Liquids were always completely removed using a soft plastic pipette (e.g., BRANDT, #612-2856) to avoid any scratching of the chip surface. In a separate tube, 10 μl of previously labeled, single-stranded DNA was dissolved in 90 μl hybridization buffer C1. The hybridization was carried out at 50⋅C and 550 rpm for 1 h. After hybridization, the ArrayStrips were washed twice using 200 μl washing buffer C2 at 45⋅C for 10 min, shaking at 550 rpm. Peroxidase-streptavidin conjugate C3 was diluted 1:100 in buffer C4. A total of 100 μl of this mixture was added to each well of the ArrayStrip and subsequently incubated at 30⋅C and 550 rpm for 10 min. Thereafter, two washing steps with 200 μl C5 washing buffer were carried out at 550 rpm at 30⋅C for 5 min. The visualization was achieved by adding 100 μl of staining substrate D1 to the ArrayStrips, and signals were detected using the ArrayMate device (Alere Technologies GmbH). Finally, an automatically generated HTML-report was provided giving information on the presence or absence of antimicrobial resistance genes and the affiliation to one of the more common species.

### Ethic statement

An Ethic Statement is not necessary. The isolates were obtained by noninvasive rectal swabs and no animal experiments were carried out for this study.

## Results

### Antimicrobial resistance genotype and phenotype

**Figure 2:**
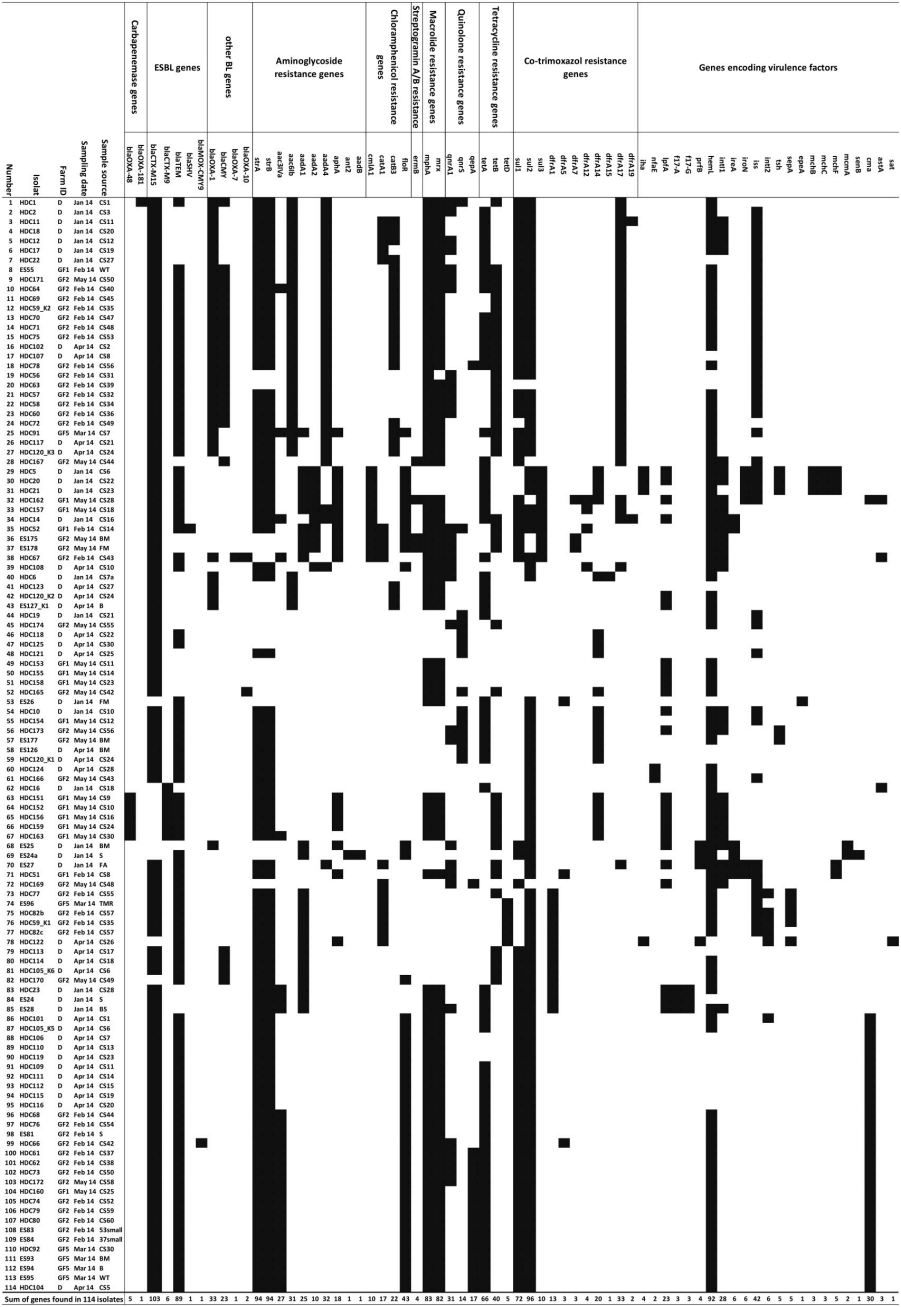
**Overview of antimicrobial resistance pattern**. Antimicrobial resistant genes of all *E. coli* isolates obtained from swab samples (healthy dairy cattle and/or environment). Also given are the farm IDs, sample sources and sampling dates. (Abbreviations: CS, rectal swab; WT, water trough; BM, bulk milk; S, soil; FM, feed mixer; FA, feed animal; BS, boot swab; B, bedding; TMR, total mixed ration).

Rectal swabs samples (*n* = 210) yielded 98 (46.6%) cultures and environmental samples (*n* = 56) yielded 16 (28.6%) cultures of putatively ESBL-producing *E. coli*. All 114 isolates were Gram-negative, motile, catalase positive, oxidase negative and indole-producing bacteria. Additionally, all isolates caused a decrease of pH and a color change of the TSI agar indicator and gas formation in the bottom of the test tube, and were therefore assigned as *E. coli*. In total, 113 (99.1%) phenotypically 3G cephalosporin-resistant isolates harbored at least one of the ESBL genes covered by the microarray (*bla*CTX-M15, *bla*CTX-M9, *bla*TEM, *bla*SHV; Figure 2). The carbapenemase gene *bla*OXA-48 was detected in five isolates (3.4%) and the carbapenemase gene *bla*OXA-181 (0.8%) was detected in one isolate (Table 1, Figure 2). These isolates showed a phenotypic resistance to imipenem and meropenem (Table 2). The total number of detected resistance genes is listed in Table 1 and an overview to each isolate is given in Figure 2. The ESBL gene *bla*CTX-M1/15 was found in 103 isolates whereas *bla*CTX-M9 was only found in 9 isolates. Consensus sequences for *bla*TEM and *bla*SHV were found in 89 and 1 isolate, respectively. For all detected beta-lactamase genotypes, the phenotype was analyzed using the VITEK-2 instrument. The results are shown in Table 2. The concordance for the carbapenem resistance and ESBL genotype was 100%. In one phenotypic ESBL positive isolate, only the narrow spectrum beta-lactamase (NSBL) gene *bla*OXA-1 was found. Due to this unexpected phenotype the concordance for the NSBL genotype was only 60%.

**Table 1:** **Antimicrobial resistance genes and their frequency in *E. coli* isolates detected by microarray**.

**Gene family**	**Genes**	**Accession No**.	**Description**	**Frequency**
Carbapenemase	*bla*OXA-48	AY236073.2	Carbapenemase, class D carbapenem hydrolyzing beta-lactamase	5
	*bla*OXA-181	JN205800.1	Carbapenemase, class D carbapenem hydrolyzing beta-lactamase	1
ESBL (extended spectrum beta- lactamase)	*bla*CTX-M1/15	X92506.1	Class A extended-spectrum-beta-lactamase	103
	*bla*CTX-M9	AF174129.3	Class A extended-spectrum-beta-lactamase	6
	*bla*TEM	Consensus	Class A beta-lactamase	89
	*bla*SHV	Consensus	Class A beta-lactamase	1
	*bla*MOX-CMY9	AF381617.1	Class C beta-lactamase	1
NSBL (narrow spectrum beta-lactamase)	*bla*OXA-1	AY458016.1	Class D beta-lactamase	33
	*bla*OXA-7	AY866525.1	Class D beta-lactamase	1
	*bla*OXA-10	J03427.1	Class D beta-lactamase	2
	*bla*CMY	AB212086.1	Class C beta-lactamase	23
Aminoglycosides	strA	EF090911.1	Aminoglycoside-3″-phosphotransferase (locus A)	94
	strB	EF090911.1	Aminoglycoside-6″-phosphotransferase	94
	aadA4	Z50802.3	Aminoglycoside adenyltransferase	32
	aac(6′)-Ib	AM283490.1	Aminoglycoside 6′-N-acetyltransferase	31
	aac(3′)-IVa	EU784152.1	Aminoglycoside 3′-N-acetyltransferase	27
	aadA1	EU704128.1	Aminoglycoside adenyltransferase	25
	aphA	AY260546.3	Aminoglycoside 3′-phosphotransferase	18
	aadA2	EU704128.1	Aminoglycoside adenyltransferase	10
	aadB	L06418.4	Aminoglycoside 2″-O-nucleotidyltransferase	1
Chloramphenicol	floR	AF252855.1	Florfenicol export protein	43
	catB3	AJ009818.1	Chloramphenicol acetyltransferase (group B)	22
	catA1	V00622.1	Chloramphenicol acetyltransferase (group A)	17
	cmlA1	EF113389.1	Chloramphenicol transporter	10
Streptogramin A/B	ermB	AB089505.1	rRNA adenine N-6-methyltransferase	4
Macrolides	mphA	AB038042.1	Macrolide 2′-phosphotransferase I	83
	Mrx	AB038042.1	Unknown (downstream to mphA)	82
Fluoroquinolones	qnrA	AY931018.1	Quinolone or fluoroquinolone resistance protein	31
	qepA	AM886293.1	Fluoroquinolone efflux pump	17
	qnrS	AM234722.1	Quinolone or fluoroquinolone resistance protein	14
Tetracycline	tetA	CP000971.1	Tetracycline resistance protein A, class A	66
	tetB	V00611.1	Tetracycline resistance protein A, class B	40
	tetD	X65876.1	Tetracycline resistance protein A, class D	5
Co-trimoxazole	sul2	DQ464881.1	Dihydropteroate synthetase type 2	96
	sul1	AJ698325.1	Dihydropteroate synthetase type 1	72
	dfrA17	AF169041.1	Dihydrofolate reductase type 17	33
	dfrA14	AJ313522.1	Dihydrofolate reductase type 14	20
	dfrA1	AJ884723.1	Dihydrofolate reductase type 1	13
	sul3	AJ459418.2	Dihydropteroate synthetase type 3	10
	dfrA12	AB154407.1	Dihydrofolate reductase type 12	4
	dfrA5	AB188269.1	Dihydrofolate reductase type 5	3
	dfrA7	AB161450.1	Dihydrofolate reductase type 7	3
	dfrA19	AJ310778.1	Dihydrofolate reductase type 19	2
	dfrA15	Z83311.1	Dihydrofolate reductase type 15	1

**Table 2:** **In detail comparison between the microarray-based genotype and the phenotype obtained by a VITEK-2 system**.

**AMR genotype detected by microarray^a^**	**Expected AMR phenotype**	**Frequency of genotype detected by microarray**	**Detected phenotype**																				**Concordance of expected AMR phenotype (%)**
			**Antibiotic tested**	**Resistant**	**Susceptible**	**Concordance (%)**	**Antibiotic tested**	**Resistant**	**Susceptible**	**Concordance (%)**	**Antibiotic tested**	**Resistant**	**Susceptible**	**Concordance (%)**	**Antibiotic tested**	**Resistant**	**Susceptible**	**Concordance (%)**	**Antibiotic tested**	**Resistant**	**Susceptible**	**Concordance (%)**	
*bla*OXA-48, *bla*CTX-M9, *bla*TEM	Carbapenem resistant	5	MEM^b^	5	0	100	IMP	5	0	100	CTX	5	0	100	CAZ	5	0	100	Amp	5	0	100	100
*bla*OXA-181, *bla*CTX-M1/15, *bla*TEM, *bla*OXA-1, *bla*CMY	Carbapenem resistant	1	MEM	1	0	100	IMP	1	0	100	CTX	1	0	100	CAZ	1	0	100	Amp	1	0	100	100
		**6**		**6**	**0**	**100**		**6**	**0**	**100**		**6**	**0**	**100**		**6**	**0**	**100**		**6**	**0**	**100**	**100**
*bla*CTX-M1/15, *bla*TEM	3G cephalosporin resistant^c^	53	MEM	0	53	100	IMP	0	53	100	CTX	53	0	100	CAZ	53	0	100	Amp	53	0	100	100
*bla*CTX-M1/15, *bla*TEM, *bla*OXA-1, *bla*CMY	3G cephalosporin resistant	16	MEM	0	16	100	IMP	0	16	100	CTX	16	0	100	CAZ	16	0	100	Amp	16	0	100	100
*bla*CTX-M1/15	3G cephalosporin resistant	12	MEM	0	12	100	IMP	0	12	100	CTX	12	0	100	CAZ	12	0	100	Amp	12	0	100	100
*bla*CTX-M1/15, *bla*OXA-1	3G cephalosporin resistant	10	MEM	0	10	100	IMP	0	10	100	CTX	10	0	100	CAZ	10	0	100	Amp	10	0	100	100
*bla*CTX-M1/15, *bla*TEM, *bla*CMY	3G cephalosporin resistant	4	MEM	0	4	100	IMP	0	4	100	CTX	4	0	100	CAZ	4	0	100	Amp	4	0	100	100
*bla*CTX-M1/15, *bla*TEM, *bla*OXA-1	3G cephalosporin resistant	3	MEM	0	3	100	IMP	0	3	100	CTX	3	0	100	CAZ	3	0	100	Amp	3	0	100	100
*bla*CTX-M1/15, *bla*CMY	3G cephalosporin resistant	1	MEM	0	1	100	IMP	0	1	100	CTX	1	0	100	CAZ	1	0	100	Amp	1	0	100	100
*bla*CTX-M1/15, *bla*MOX-CMY9, *bla*TEM	3G cephalosporin resistant	1	MEM	0	1	100	IMP	0	1	100	CTX	1	0	100	CAZ	1	0	100	Amp	1	0	100	100
*bla*CTX-M1/15, *bla*TEM, *bla*OXA-7	3G cephalosporin resistant	1	MEM	0	1	100	IMP	0	1	100	CTX	1	0	100	CAZ	1	0	100	Amp	1	0	100	100
*bla*CTX-M1/15, *bla*TEM, *bla*SHV	3G cephalosporin resistant	1	MEM	0	1	100	IMP	0	1	100	CTX	1	0	100	CAZ	1	0	100	Amp	1	0	100	100
*bla*CTX-M9	3G cephalosporin resistant	1	MEM	0	1	100	IMP	0	1	100	CTX	1	0	100	CAZ	1	0	100	Amp	1	0	100	100
*bla*TEM (consensus), *bla*CMY	3G cephalosporin resistant	1	MEM	0	1	100	IMP	0	1	100	CTX	1	0	100	CAZ	1	0	100	Amp	1	0	100	100
*bla*TEM (consensus)	3G cephalosporin resistant	3	MEM	0	3	100	IMP	0	3	100	CTX	3	0	100	CAZ	3	0	100	Amp	3	0	100	100
		**107**		**0**	**107**	**100**		**0**	**107**	**100**		**107**	**0**	**100**		**107**	**0**	**100**		**107**	**0**	**100**	**100**
*bla*OXA-1	NSBL	1	MEM	0	1	100	IMP	0	1	100	CTX	1	0	0	CAZ	1	0	0	Amp	1	0	100	60
		**1**		**0**	**1**	**100**		**0**	**1**	**100**		**1**	**0**	**0**		**1**	**0**	**0**		**1**	**0**	**100**	**60**
aac3lVa	Aminoglycoside resistant	20	CN	20	0	100	TOB	20	0	100													100
aac6lb	Aminoglycoside resistant	5	CN	5	0	100	TOB	5	0	100													100
aadA1	Aminoglycoside resistant	11	CN	1	10	90	TOB	1	10	90													90
aadA1, aac3lVa	Aminoglycoside resistant	1	CN	1	0	100	TOB	1	0	100													100
aadA2, aadA4	Aminoglycoside resistant	1	CN	0	1	0	TOB	0	1	0													0
aadA4	Aminoglycoside resistant	2	CN	1	1	50	TOB	1	1	50													50
aadA4, aac6lb	Aminoglycoside resistant	25	CN	22	3	88	TOB	25	0	100													94
aadB, aadA1	Aminoglycoside resistant	1	CN	0	1	0	TOB	0	1	0													0
aphA	Aminoglycoside resistant	6	CN	0	6	100	TOB	0	6	100													100
aphA, aadA1	Aminoglycoside resistant	4	CN	0	4	100	TOB	0	4	100													100
aphA, aadA1, aadA2	Aminoglycoside resistant	6	CN	6	0	100	TOB	0	6	0													50
aphA, aadA1, aadA4	Aminoglycoside resistant	1	CN	1	0	100	TOB	1	0	100													100
aphA, aadA1, aadA4, aac6lb, aac3lVa	Aminoglycoside resistant	1	CN	1	0	100	TOB	1	0	100													100
aphA, aadA1, aadA2, aadA4	Aminoglycoside resistant	1	CN	1	0	100	TOB	1	0	100													100
n.d.	Aminoglycoside susceptible	29	CN	9	20	69	TOB	9	20	69													69
		**114**		**68**	**46**	**73**		**65**	**49**	**67**													**77**
tetA	Tetracycline resistant	55	TE	55	0	100	TGC	1	54	98													99
tetA, tetB	Tetracycline resistant	14	TE	14	0	100	TGC	0	14	100													100
tetB	Tetracycline resistant	26	TE	26	0	100	TGC	0	26	100													100
tetD	Tetracycline resistant	6	TE	6	0	100	TGC	0	6	100													100
n.d.	Tetracycline susceptible	13	TE	4	9	0	TGC	0	13	100													50
		**114**		**105**	**9**	**80**		**1**	**113**	**100**													**90**
qepA	Fluoroquinolone resistant	14	CIP	14	0	100	MXF	14	0	100													100
qepA, qnrA1	Fluoroquinolone resistant	2	CIP	2	0	100	MXF	2	0	100													100
qnrA1	Fluoroquinolone resistant	31	CIP	30	1	97	MXF	30	1	97													97
qnrB	Fluoroquinolone resistant	1	CIP	1	0	100	MXF	1	0	100													100
qnrS	Fluoroquinolone resistant	10	CIP	3	7	30	MXF	8	2	80													55
qnrS, qnrA1	Fluoroquinolone resistant	4	CIP	2	2	50	MXF	3	1	75													63
n.d.	Fluoroquinolone susceptible	52	CIP	34	18	35	MXF	34	18	35													35
		**114**		**86**	**28**	**73**		**92**	**22**	**84**													**79**
sul1, dfrA17	Co-trimoxazol resistant	1	STX	1	0	100																	100
sul1, sul2, dfrA1	Co-trimoxazol resistant	6	STX	6	0	100																	100
sul1, sul2, dfrA17	Co-trimoxazol resistant	22	STX	22	0	100																	100
sul1, sul2, dfrA17, dfrA19	Co-trimoxazol resistant	1	STX	1	0	100																	100
sul1, sul2, dfrA5	Co-trimoxazol resistant	1	STX	1	0	100																	100
sul1, sul2, sul3, dfrA12	Co-trimoxazol resistant	1	STX	1	0	100																	100
sul1, sul2, sul3, dfrA12, dfrA17	Co-trimoxazol resistant	1	STX	1	0	100																	100
sul1, sul2, sul3, dfrA17	Co-trimoxazol resistant	1	STX	1	0	100																	100
sul1, sul3, dfrA5, dfrA7, dfrA14, dfrA15, dfrA17	Co-trimoxazol resistant	1	STX	1	0	100																	100
sul1, sul3, dfrA7	Co-trimoxazol resistant	1	STX	1	0	100																	100
sul1, sul3, dfrA7, dfrA17	Co-trimoxazol resistant	1	STX	1	0	100																	100
sul2, dfrA1	Co-trimoxazol resistant	7	STX	7	0	100																	100
sul2, dfrA12, dfrA17	Co-trimoxazol resistant	1	STX	1	0	100																	100
sul2, dfrA14	Co-trimoxazol resistant	14	STX	14	0	100																	100
sul2, dfrA14, dfrA15	Co-trimoxazol resistant	1	STX	1	0	100																	100
sul2, dfrA17	Co-trimoxazol resistant	4	STX	4	0	100																	100
sul2, dfrA5	Co-trimoxazol resistant	2	STX	2	0	100																	100
sul2, sul3, dfrA14	Co-trimoxazol resistant	2	STX	2	0	100																	100
sul3, dfrA14	Co-trimoxazol resistant	1	STX	1	0	100																	100
sul1	Co-trimoxazol susceptible	1	STX	1	0	0																	0
sul2	Co-trimoxazol susceptible	3	STX	1	2	66																	66
sul1, sul2	Co-trimoxazol susceptible	30	STX	29	1	3																	3
sul1, sul2, sul3	Co-trimoxazol susceptible	1	STX	1	0	0																	0
dfrA14	Co-trimoxazol susceptible	1	STX	1	0	0																	0
dfrA17	Co-trimoxazol susceptible	1	STX	1	0	0																	0
n.d.	Co-trimoxazol susceptible	8	STX	0	8	100																	100
		**114**		**103**	**11**	**80**																	**80**
																	**Overall Concordance (%)**						**83.5**

a A genotype was defined as a group of genes which have been described to confer resistance to a family of antibiotics (e.g., the genotype “aac6, aac6ib, aadA1” confers resistance to aminoglycosides). The phenotype was detected by a VITEK-2 system.

b AK amikacin, CN gentamicin, TOB tobramycin, FEP cefepime, CTX cefotaxime, CAZ ceftazidime, MEM meropenem, IMP imipenem, TE tetracycline, TGC tigecycline, STX co-trimoxazole, CIP ciprofloxacin, MXF moxifloxacin.

c 3G cephalosporin – 3rd generation cephalosporin.

Nine different aminoglycoside resistance genes were detected by the *E. coli* PanType AS-1 kit (Table 1). The combinations of these genes resulted in 14 different genotypes, whereas the most prevalent genotype was *aac(6*′*)-Ib* in combination with *aadA4* (*n* = 25). All isolates harboring this genotype were resistant to tobramycin, but three of them were susceptible to gentamicin (Table 2). Therefore, the concordance between genotype and phenotype was 94%. Five isolates, which harbored only *aac(6*′*)-Ib*, were resistant to all tested aminoglycosides. The second most frequent genotype was *aac(3)-lVa* (*n* = 20). All isolates harboring this gene were resistant to gentamicin and tobramycin and corresponded to 100% of the expected phenotype (Table 2). Six isolates harboring the gene *aphA* were susceptible to tobramycin and gentamicin. The detected phenotype corresponded 100% with the genotype, as the enzyme AphA does not mediate resistance against both aminoglycoside antibiotics tested (Ramirez and Tolmasky, 2010).

The most prevalent genotype for fluoroquinolone resistance was *qnrA1* followed by *qepA*. One isolate harboring *qnrA1* was sensitive to both quinolone antibiotics tested (97.0% concordance), but all isolates with detected *qepA* gene were resistant (100% concordance). Overall, 86 of 114 isolates were resistant to ciprofloxacin and 92 against moxifloxacin. Only in 56 ciprofloxacin resistant isolates a corresponding genotype was detected. Similar results were observed for the 92 moxifloxacin resistant isolates, where only in 68 isolates a corresponding genotype was detected. The overall concordance of the detection of genes mediating fluoroquinolone resistance with phenotypic resistance was 79.0% (Table 2).

Resistance to co-trimoxazole is associated with *sul* and *dfrA* genes. All isolates with this gene combination were resistant to co-trimoxazole (Table 2). From 45 isolates without this gene combination, 34 were resistant. Therefore, the concordance of genotype and phenotype was 80.0%.

Of 114 isolates, one was resistant to fosfomycin. Such resistance is caused by mutations in ubiquitous genes (e.g., *murA* or *glpT*) or the loss of entire genes (e.g., *uhpA*) rather than by acquisition of distinct resistance markers and therefore the genotypes were not included into the test panel (Takahata et al., 2010; Li et al., 2015).

In summary, the overall concordance among all genotypes to expected phenotypes was 82.6% (Table 2).

### Serotyping

**Figure 3:**
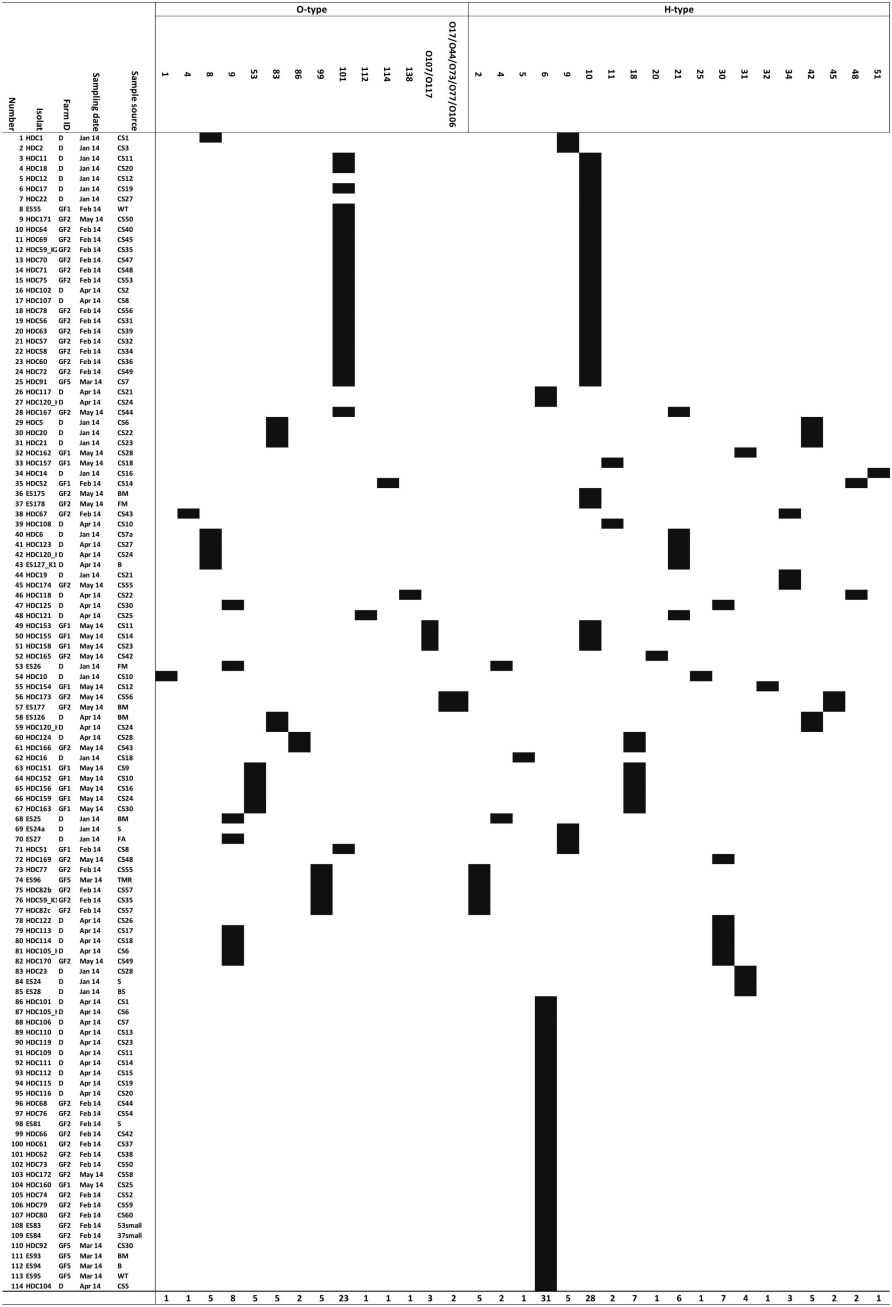
**Overview of microarray-based serotyping**. Serotype of all *E. coli* isolates obtained from swab samples (healthy dairy cattle and/or environment). Also given are the farm IDs, sample sources and sampling dates. (Abbreviations: CS, rectal swab; WT, water trough; BM, bulk milk; S, soil; FM, feed mixer; FA, feed animal; BS, boot swab; B, bedding; TMR, total mixed ration).

For all 114 ESBL-producing *E. coli* isolates, the O- and H-types were identified using the *E. coli* SeroGenotyping AS-1 kit (Figure 3). For 63 (55.3%) isolates, genes encoding both O and H antigens were detected and for 51 isolates (44.7%) only the gene encoding the H antigen could be detected. The most prevalent serotype was O101:H10. This serotype was found in locations D, GF1, and GF2 and isolated mainly from rectal swabs, as one isolate belonging to this group was found in a water trough. The AMR genotype for O101:H10 isolates was rather uniform (Figure 3). Isolates of serotype O53:H18 with the carbapenemase gene *bla*OXA-48 were only found in farm GF1 and were isolated from rectal swabs. All isolates belonging to this group were identical with regard to their phenotype and genotypes (Table 2, Figures 2, 3). The serotype O8:H9 with *bla*OXA-181 was found only once in farm D from a rectal swab. For isolates where only the H-antigen H6 was found, a very similar AMR genotypes was detected. Such isolates were found in all investigated farms and sample types.

## Discussion

In 2014, four dairy farms in northern Egypt were investigated for ESBL-producing *E. coli*. In total, 210 clinically healthy dairy cattle were sampled using rectal swabs. Additionally, 56 environmental swabs were taken from different stall objects and screened for multi-drug resistant bacteria. All swabs were pre-cultured on Brilliance™ ESBL Agar, and in 114 of 266 samples (42.8%), ESBL-producing *E. coli* were detected. To analyze the underlying molecular AMR mechanism, all 114 isolates were genotyped using the multiplex microarray technique. The most frequently detected gene which mediated resistance against 3G cephalosporins was *bla*CTX-M1/15 (90.4%). The genes *bla*CTX-M9 (5.3%) and *bla*TEM (78%), which also mediate resistance to 3G cephalosporins, were also detected. However, both were usually found in combination with *bla*CTX-M1/15. *Bla*CTX-M9 was related to resistance to 3G cephalosporins in just one isolate as well as *bla*TEM in four isolates. A comparison to data similar to data from this study is difficult due to missing reports from Egypt. Recently, two reports from Germany are known describing the prevalence of ESBL-producing bacteria in livestock of healthy animals (Schmid et al., 2013; Dahms et al., 2015). Like in the present study, in both reports the most prevalent ESBL group was CTX-M. Schmid et al. (2013) collected a total of 598 samples that yielded 196 ESBL-producing *E. coli* (32.8%). The high percentage of ESBL-producing *E. coli* in healthy animals shows the high zoonotic risk for people working in close contact to animals. With this background Dahms et al. (2015) investigated different farms for ESBL-producing bacteria in samples collected from livestock and also from farm workers. In total, 70.6% of the tested farms and 5.8% of the farm workers were positive for ESBL-producing bacteria. In contrast, a study from Burgundy in France in 2012 showed only a low prevalence, of about 5%, of ESBL-producing bacteria in feces samples from different farms (Hartmann et al., 2012).

Due to the preselection of all samples on Brilliance™ ESBL agar, carbapenem resistant isolates were also retrieved. In six isolates, carbapenemase genes were detected using the CarbDetect AS-1 kit. In five isolates from a stall on farm GF1, *bla*OXA-48 was found. These isolates were phenotypically and genotypically identical. Interestingly, another isolate was found containing *bla*OXA-181, a gene belonging to the *bla*OXA-48-like family. Both genes are also widely distributed in human pathogens that cause hospital-acquired infection (Poirel et al., 2012). Carbapenems are last-line antibiotics with a broad spectrum and a high efficacy, and are stable against ESBLs. As they can only be administered intravenously, carbapenems are used exclusively in the clinical environment, and are not known to be used in animal husbandry. While carbapenemase-producing *Enterobacteriaceae* (CPE) are mostly described in hospitalized humans (Nordmann et al., 2011; Abdallah et al., 2015b), the current study shows conclusively that such CREs are also found in farm animals including apparently healthy dairy cattle. The detection of carbapenem resistant isolates in such an environment and the threat that such multi-drug resistance bacteria ends up in consumer food (e.g., milk or dairy products), raises serious concerns about public health. Carbapenemase-producing isolates have been detected in poultry farms (Abdallah et al., 2015a), but there are to date no reports describing finding of these multi-drug resistant bacteria in dairy cattle farms in Egypt.

Given that the majority of samples that contained ESBL/carbapenemase-producing bacteria originated from rectal swabs, this raises the question of how the potentially contaminated feces are disposed of. Normally, dung is used as fertilizer in agriculture. Via this route, multi-drug resistant pathogens might get into the food chain, either directly through consumption of meat, or indirectly from cattle grazing on fertilized pasture. Another major problem raises up in this context, the resistance genes described in this paper are usually found on plasmids (Chanawong et al., 2001; Paterson and Bonomo, 2005; Duan et al., 2006; Wittum et al., 2010; Brolund, 2014; Hansen et al., 2014; Valentin et al., 2014) and such mobile elements can be easily transferred to and between environmental bacteria (Aminov, 2011; Berglund, 2015), as well as to other human pathogens (Pitout et al., 2015). This poses a high risk to the environment, and the human population.

## Conclusion

To the best of our knowledge, this study is the first report which analyses the prevalence of ESBL-producing *E. coli* in Egyptian dairy farms based on genotyping and phenotyping data. The high percentage of ESBL-positive isolates and even of carbapenemase-producing bacteria was alarming given the relevance of 3G cephalosporins and carbapenems in modern medicine. Additionally, the isolates had a highly diverse genetic background with regard to serotype, virulence and antimicrobial resistance markers (Figures 2, 3). Experiments showed a high degree of concordance between genotype and phenotype.

Strict hygiene measures are mandatory to control the spread, the transmission dynamics and potential zoonotic risk factors of ESBL- and carbapenemase-producing bacteria in dairy farms.

## Author contributions

SB, HE, and HH conceived of the study, and participated in its design and coordination. SB, IE, and DW carried out the genotyping and serotyping. SB and IE carried out the antimicrobial resistance pattern by VITEK-2. MA and HE participated in sampling. HE, HH, and MA participated in preliminary design as well as bacteriological analysis of part of the study. SB, SM, HE, HH, and RE drafted the manuscript. All authors read and approved the final manuscript.

### Conflict of interest statement

SB, DW, SM, IE, and RE are employees of Alere Technologies GmbH, the company that manufactures the microarrays also used in this study. This has no influence on study design, data collection and analysis, and this does not alter the authors' adherence to all the Frontiers policies on sharing data and materials. The other authors declare that the research was conducted in the absence of any commercial or financial relationships that could be construed as a potential conflict of interest.
